# The first complete mitochondrial genome of *Zoodes fulguratus* (Gahan 1906) (Coleoptera: Cerambycidae: Cerambycinae) and its phylogeny

**DOI:** 10.1080/23802359.2021.1935354

**Published:** 2021-06-07

**Authors:** Sam Pedro Galilee Ayivi, Yao Tong, Jia-Yin Guan, Kenneth B. Storey, Dan-Na Yu, Jia-Yong Zhang

**Affiliations:** a College of Chemistry and Life Science, Zhejiang Normal University, Jinhua, PR China; b Department of Biology, Carleton University, Ottawa, Canada; c Key Lab of Wildlife Biotechnology, Conservation and Utilization of Zhejiang Province, Zhejiang Normal University, Jinhua, Zhejiang Province, PR China

**Keywords:** *Zoodes fulguratus*, Hesperophanini, Cerambycidae, mitochondrial genome, phylogeny

## Abstract

*Zoodes fulguratus* is a common species of Cerambycidae, reported from Vietnam, Nepal, Laos, Burma, and China. To date, no mitochondrial genomes of the genus *Zoodes* have been reported. In this study, we sequenced and analyzed the first mitochondrial genome of *Z. fulguratus* to discuss its phylogenetic relationship within the subfamily Cerambycinae. This mitochondrial genome showed the typical insect gene arrangement: a circular molecule of 15,885 bp long with 13 protein-coding genes, two ribosomal RNA genes (rRNAs), and 22 transfer RNA genes (tRNAs). The AT content of the whole mitogenome was 74.2% with a high asymmetric nucleotide presentation revealed by a positive AT-skew (0.067) and a negative GC-skew (–0.178), whereas the AT content of the A + T rich region was 80%. The Maximum likelihood (ML) and Bayesian inference (BI) phylogenetic analyses showed that *Z. fulguratus* is a sister clade of *Gnatholea eburifera.*

*Zoodes fulguratus* (Gahan 1906) (Coleoptera: Cerambycidae: Cerambycinae: Hesperophanini) is one of the common *Zoodes* species present in China and also occurs in Vietnam, Nepal, Laos, and Burma (Vives and Ghate 2015; Tavakilian and Chevillotte 2019). However, it is one of the lesser represented members of the family Cerambycidae. The phylogenetic relationships of Cerambycidae remain debated and new mitogenomes are needed for phylogenetic analysis (Kim et al. 2009; Wang, Dai, et al. 2019; Nie et al. 2021). To date, no mitochondrial genomes of the genus *Zoodes* have been reported. In this study, we sequenced and analyzed the first mitochondrial genome of *Z. fulguratus* to discuss its phylogenetic relationship within Cerambycinae.

The sample used in this study was collected from Yingjiang County, Yunnan Province, China (N 24^°^45′, E 97^°^42′, 25 July 2018) and identified by Dr. JY Zhang. The specimen under the voucher number: YNYJ-20180725 was preserved in a −40 °C freezer and deposited at the Animal Specimen Museum, College of Life Sciences and Chemistry, Zhejiang Normal University, China (sky.zjnu.edu.cn, JY Zhang, zhang3599533@163.com). Total genomic DNA was extracted from dissected thorax muscle using an Ezup Column Animal Genomic DNA Purification Kit (Sangon Biotech Company, Shanghai, China) and stored in the Zhang laboratory. Universal primers (Wang, Dai, et al. 2019) and nine specific primers were used for PCR and LA PCR to amplify the whole mitogenome. The mitochondrial genome was deposited in the NCBI with accession number MW858149.

The complete mitochondrial genome of *Z. fulguratus* was 15,885 bp in length, which is the longest number of nucleotide bases reported to date in subfamily Cerambycinae. It encoded of 37 genes including 13 protein-coding genes (PCGs), 22 transfer RNAs, 2 ribosomal RNAs genes, and 1 non-coding A + T rich region (D-loop region). The total length of the PCGs was 11,447 bp and all genes showed a negative AT-skew. The start codons of the PCGs in *Z. fulguratus* were ATG (in ND2, ATP6, COX3, ND4, ND4L, and Cyt b), ATA (in ND3, ND5, ND6), ATT (in COX2, ATP8, and ND1), and ATC (in COX1). TAA was the most commonly used stop codon (in ND2, COX1, ATP6, COX3, ND3, ND4, ND4L, and ND6) followed by TAG (in ATP8, Cyt b, ND1) and the incomplete stop codon T- (in COX2, and ND5). The length of 22 tRNAs, two rRNAs and D-loop region was 1449, 2074, and 1234 bp, respectively. Most of the 22 transfer RNAs have a classical cloverleaf structure except tRNA-S1 without DHU arm, which is common in metazaon mitogenomes (Zhang et al. 2021). The 16S RNA and 12S RNA genes were located between tRNA-Leu and tRNA-Val, tRNA-Val and the AT-rich region, respectively. The AT-rich region was between 12S RNA and tRNA-Ile, which had two tandem repeat nucleotide sequences (52 and 75 bp). In the whole mitochondrial genome, we found 17 overlapping areas each ranging from 1 to 8 bp and 7 intergenic spacers each ranging from 1 to 18 bp. The total length of the overlapped and intergenic region was 51 and 32 bp, respectively. The gene arrangement was identical to the ancestral insect gene order pattern. The whole mitogenome sequence base content was 39.6% A, 34.6% T, 15.2% C, 10.6% G with A + T = 74.2% and G + C = 25.8%.

In order to determine phylogenetic relationships within Cerambycinae, we used 25 species including *Z. fulguratus* with 22 other species from Cerambycinae and one species each from Prioninae and Laminae subfamilies as outgroups (Wang, Lan, et al. 2019; Dai et al. 2020), all downloaded from NCBI. The nucleotide sequences of the 13 PCGs were aligned by multiple alignment using fast Fourier transform (MAFFT) (Katoh and Standley 2013). The aligned PCGs data of the different species were concatenated using Mesquite software (Maddison and Maddison 2019) and the best-fit substitution model (GTR + I+G) for phylogenetic analyses was determined using PartitionFinder version 2.2.1 (Lanfear et al. 2012). Maximum likelihood (ML) and Bayesian inference (BI) were used to construct the phylogenetic tree by RAxML (Stamatakis 2014) and MrBayes (Huelsenbeck and Ronquist 2001), respectively. Our phylogenetic analyses showed that *Z. fulguratus* was the sister clade of *Gnatholea eburifera* and that Callidiini and Hesperophanini were not monophyletic groups (Figure 1) consistent with the results of Nie et al. (2021). In the phylogenetic relationships of Cerambycinae, Obriini is the basal clade among Cerambycinae and there were two clades formed in the remain Cerambycinae species. One clade was formed by the clade of (((((*Gnatholea eburifera* + *Z. fulguratus*) (Hesperophanini) + Trachyderini) + *Pyrrhidium sanguineum* (Callidiini)) + (Callidiopini + *Trichoferus campestris* (Hesperophanini)) + ((Clytini + Tillomorphini) + (*Semanotus bifasciatus* (Callidiini) + Achrysonini))); the other clade was formed by the clade of (((Callichromatini + Xystrocerini) + Molorchini) + Cerambycini). Cerambycinae is such a huge subfamily which contains 119 tribes (Tavakilian and Chevillotte 2019), new mitochondrial genomes of Cerambycinae are needed to further understand the phylogenetic relationships within Cerambycinae.

**Figure 1.**
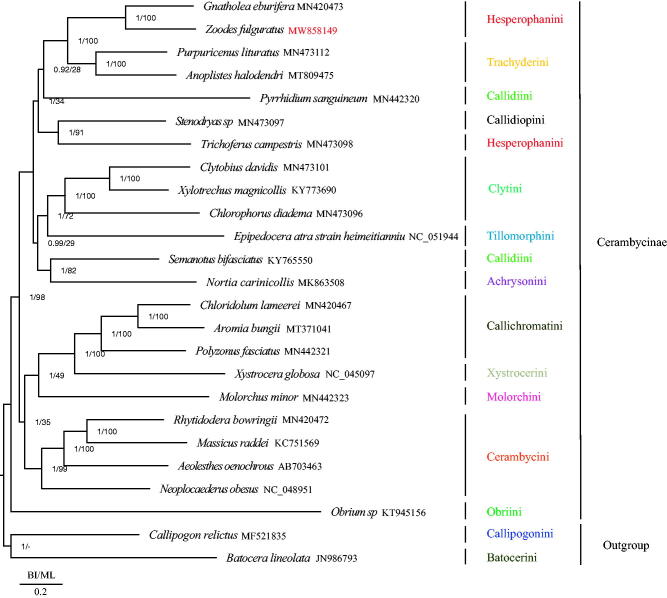
Phylogenetic tree of the relationships among 25 species of Cerambycidae including *Zoodes fulguratus* based on the nucleotide dataset of the 13 mitochondrial protein-coding genes. Numbers around the nodes are the posterior probabilities of BI (left) and the bootstrap values of ML (right). The GenBank numbers and tribe of all species are shown in the figure.

## Data Availability

The genome sequence data that support the findings of this study are openly available in GenBank of NCBI at (https://www.ncbi.nlm.nih.gov/) under the accession no. MW858149.
